# Poly(ethylene glycol) Methyl Ether Acrylate-Grafted Chitosan-Based Micro- and Nanoparticles as a Drug Delivery System for Antibiotics

**DOI:** 10.3390/polym16010144

**Published:** 2024-01-02

**Authors:** Corina-Lenuța Logigan, Christelle Delaite, Marcel Popa, Elena Simona Băcăiță, Crina Elena Tiron, Cristian Peptu, Cătălina Anișoara Peptu

**Affiliations:** 1 Department of Natural and Synthetic Polymers, Faculty of Chemical Engineering and Environmental Protection “Cristofor Simionescu”, “Gheorghe Asachi” Technical University of Iasi, Bld. Prof. Dr. Doc. Dimitrie Mangeron Street, No. 73, 700050 Iasi, Romania; savincorina@yahoo.com (C.-L.L.); marpopa@ch.tuiasi.ro (M.P.); 2 Laboratory of Photochemistry and Macromolecular Engineering, Institute J.B. Donnet, University of Haute Alsace, 68100 Mulhouse, France; christelle.delaite@uha.fr; 3 Faculty of Medical Dentistry, “Apollonia” University of Iasi, Pacurari Street, 11, Iasi 6600, Romania Muzicii Street, No. 2, 700511 Iasi, Romania; 4 Academy of Romanian Scientists, Ilfov Street, No. 3, Sector 5, 050094 Bucharest, Romania; 5 Department of Physics, Faculty of Machine Manufacturing and Industrial Management, “Gheorghe Asachi” Technical University of Iasi, Bld. Prof. Dr. Doc. Dimitrie Mangeron Street, No. 73, 700050 Iasi, Romania; elena-simona.bacaita@academic.tuiasi.ro; 6 Regional Institute of Oncology, General Henri Mathias Berthelot Street, 2–4, 700483 Iasi, Romania; transcendctiron@iroiasi.ro; 7 “Petru Poni” Institute of Macromolecular Chemistry, Aleea Grigore Ghica Voda, 41A, 700487 Iasi, Romania; cristian.peptu@icmpp.ro

**Keywords:** micro/nanoparticles, poly(ethylene glycol) methyl ether acrylate, chitosan, reverse emulsion, interpenetrating network, levofloxacin, ciprofloxacin, biomedical applications

## Abstract

Nanotechnology is the science of creating materials at the nanoscale by using various devices, structures, and systems that are often inspired by nature. Micro- and nanoparticles (MPs, NPs) are examples of such materials that have unique properties and can be used as carriers for delivering drugs for different biomedical applications. Chitosan (CS) is a natural polysaccharide that has been widely studied, but it has a problem with low water solubility at neutral or basic pH, which limits its processability. The goal of this work was to use a chemically modified CS with poly(ethylene glycol) methyl ether acrylate (PEGA) to prepare CS micronic and submicronic particles (MPs/NPs) that can deliver different types of antibiotics, respectively, levofloxacin (LEV) and Ciprofloxacin (CIP). The particle preparation procedure employed a double crosslinking method, ionic followed by a covalent, in a water/oil emulsion. The studied process parameters were the precursor concentration, stirring speeds, and amount of ionic crosslinking agent. MPs/NPs were characterized by FT-IR, SEM, light scattering granulometry, and Zeta potential. MPs/NPs were also tested for their water uptake capacity in acidic and neutral pH conditions, and the results showed that they had a pH-dependent behavior. The MPs/NPs were then used to encapsulate two separate drugs, LEV and CIP, and they showed excellent drug loading and release capacity. The MPs/NPs were also found to be safe for cells and blood, which demonstrated their potential as suitable drug delivery systems for biomedical applications.

## 1. Introduction

Chitosan (CS) is a natural polysaccharide that is widely used as a base material for polymeric nanoparticles. Chitosan has many desirable properties, such as being biocompatible, biodegradable, non-toxic, and mucoadhesive. It also has many reactive amine groups that allow it to be modified physically or chemically. Numerous studies have been conducted with the aim of modifying chitosan (CS) to enhance its characteristics, including solubility, mucoadhesion, and stability, thereby broadening its potential applications [1]. CS or CS derivative-based micro/nanoparticle (MPs/NPs) systems are one of the most popular controlled release systems for the active principle [2]. They have many advantages, such as a small size, large surface area, high drug encapsulation/absorption capacity at the cellular/intracellular level, prolonged and sustained drug release, the possibility to target the drug at diseased tissues, the protection of the active principle from enzymatic degradation (e.g., peptidases and nucleases), biocompatibility, non-toxicity and long blood circulation time [3,4,5]. The properties of chitosan micro/nanoparticles (CS-MPs/NPs) can be adjusted depending on the methods and parameters used to prepare them. This makes them suitable for different biomedical applications, such as non-parenteral drug delivery for treating cancer [6], gastrointestinal diseases [7], pulmonary diseases [8], drug delivery to the brain [9], and ocular infections [10]. To modulate the interactions of MPs/NPs with the biological environment, they can be functionalized on the surface or embedded in a polymer matrix. This can change their physicochemical properties, increase their biocompatibility and uptake, enhance their drug-loading capacity, and improve their solubility and stability in different biological media [11]. One of the most common methods to prepare CS-based MPs/NPs is the ionotropic gelation method [12]. This method uses electrostatic interactions between the amine groups of CS and negatively charged crosslinking agents to form polymeric materials with a simple and flexible network [13]. Chitosan particles as potential carriers for controlled drug delivery ionically crosslinked with sodium tripolyphosphate (TPP) have been used in many studies [14]. However, CS/TPP complexes can dissociate into free chitosan chains when exposed to a physiological pH. This means that the crosslinking process is reversible and that the nanoparticles have limited practical applications [15]. A possible solution to this problem is to add another covalent crosslinking step [6,10,16,17] in order to preserve the particle’s shape and prevent premature dissociation. Chitosan has free amine and hydroxyl groups in its structure, which enable various chemical crosslinking strategies. The free amine groups of CS can also be covalently crosslinked with aldehydes to produce a more rigid and robust nanoparticle structure, which also gives them stability under physiological conditions [6,10,16,17,18,19]. CS-based nanoparticles made by emulsification and dual crosslinking ionic and covalent have been reported in the literature. Both types of crosslinkers (e.g., ionic—sodium sulfate and covalent—GA) are important for making MPs/NPs with enhanced stability [6,10,16,20,21,22]. However, adjusting and optimizing the ionic/covalent crosslinking process for a specifically modified chitosan derivative represents a challenging task.

TPP is a common ionic crosslinker for making CS particles, as many studies have shown. However, sodium sulfate as an ionic crosslinker for CS-based MPs/NPs has been rarely used in the literature. Our research group has used double crosslinked (e.g., sodium sulfate and GA) CS-gelatin particles for eye delivery applications. Thus, non-toxic particles loaded with adrenalin were tested on animals and a human volunteer, revealing excellent eye adhesion properties without causing irritation and effectively releasing the drug to clear the eye congestion [16].

Enhancing the bioavailability of dexamethasone (DEX), potentially improving its therapeutic efficacy in ocular applications, was attributed to the higher permeability and mucoadhesive nature of CS. The obtained results of Pepic et al. showed that CS based prepared polymeric micelles presented excellent ocular bioavailability with two- to four-fold increases compared to standard DEX suspension [10]. Jatariu et al. made CS and gelatin-based NPs by both ionic and covalent crosslinking in a reverse emulsion. The NPs were marked with fluorescein and tested in vivo. The results showed that the NPs had better penetration by an intravenous route than the intraperitoneal route and that the NPs accumulated mostly in the liver and least in the testicles [16]. Alupei et al. made NPs based on a folic acid-CS derivative by emulsification and double crosslinking method for cancer therapy. The NPs based on folic acid-CS derivative had a higher ability to bind and attach to the tumor cells. The NPs were also stable, non-toxic, and had a suitable size for intravenous administration [23]. Wulandari et al. synthesized CS-coated iron oxide nanoparticles by ex situ co-precipitation method as a candidate for drug delivery. The NPs were coated and crosslinked with CS and tripolyphosphate/sulfate as ionic crosslinkers. The results showed that the combination of tripolyphosphate and sulfate as crosslinking agents produced smaller and spherical chitosan-Fe_3_O_4_ particles [24]. Different studies showed that CS-based NPs could encapsulate and release different types of drugs at a constant rate, which showed the potential of CS as a sustained-release matrix polymer [10,16,17,23,25,26]. Levofloxacin and ciprofloxacin are two antibiotics from the second-generation quinolones. They have good properties, such as good oral absorption, good tissue diffusion, and good permeation into phagocytic cells. They are also widely used and prescribed for treating various infections of the urinary tract, skin, intraabdominal, pelvic infections, and chronic bronchitis [27,28,29,30,31,32,33]. Nanocarriers can help overcome biological barriers, protect and deliver drugs to target cells, and improve drug characteristics [31]. It is important to note that both LEV and CIP can cause side effects, and reducing the administrated dose through their formulation as micro/nanoparticle systems may contribute to the reduction of systemic toxicity. Antibiotics can be protected from environmental degradation and delivered more effectively loaded into polymeric particles. The prepared MPs/NPs system can penetrate dense tissues and hard-to-reach cells that harbor bacteria. They could also target specific tissues, cells, or bacteria or release the antibiotic only when they encounter the infection site. This way, the antibiotic can reach the infection site in higher concentrations while reducing the dose and frequency of administration [32].

CS-PEGA nanoparticles were used to improve the LEV and CIP nanocarrier characteristics. The studies showed that LEV loaded in CS-PEGA MPs/NPs had a slow release rate, which could reduce the frequency of administration and the side effects of the drug [33]. Ciprofloxacin-loaded CS MPs/NPs were also developed and evaluated for their physicochemical properties. The studies showed that CIP loaded in CS MPs/NPs had better antibacterial activity, inhibiting the growth of two types of Gram-positive and Gram-negative bacteria [34].

The current work aims the optimization the particle preparation process in order to adapt the ionic gelation with Na_2_SO_4_ for the chitosan grafted poly(ethylene glycol) methyl ether acrylate (CS-PEGA) derivative. Further, the ionic gelation processed particles are protected through a covalent crosslinking reaction in a water/oil emulsion.

The microparticles (MPs) and nanoparticles (NPs) underwent characterization to assess their structure, size, shape, and swelling properties, revealing pH-dependent behavior. Additionally, these MPs/NPs were subjected to testing to evaluate their potential as drug delivery devices, hemocompatibility, and cytotoxicity. Thus, MPs/NPs were loaded with LEV or CIP as model drugs, and the results showed a prolonged drug release, which recommends them as an alternative for biomedical applications.

Our previous study demonstrated the viability of the method by obtaining particulate formulations crosslinked ionically with sodium tripolyphosphate (TPP) and covalently with glutaraldehyde (GA). In the present study, we were interested in investigating the change of the ionic crosslinker from TPP to sodium sulfate and its influence on the final products. The results of this investigation will provide valuable insights into the suitability of the CS-PEGA polymer for particle production under these specific conditions. It will also shed light on the unique properties of the system prepared with these parameters. This could potentially lead to the development of more efficient and effective particle-based applications in various fields.

## 2. Materials and Methods

### 2.1. Materials

Chitosan (CS, low molecular weight, 88% hydrolysis degree, Sigma Aldrich, Saint Louis, MO, USA); Poly(ethylene glycol) methyl ether acrylate (PEGA, Mn = 480, Sigma Aldrich, Saint Louis, MO, USA); acetic acid (CH_3_COOH, 99%, Sigma Aldrich, Saint Louis, MO, USA); glutaraldehyde (GA, Sigma Aldrich, 25% aqueous solution, Saint Louis, MO, USA); sodium sulfate (Na_2_SO_4_, Sigma Aldrich, Saint Louis, MO, USA); levofloxacin (LEV, Sigma Aldrich, Saint Louis, MO, USA); ciprofloxacin (CP, Sigma Aldrich, Saint Louis, MO, USA); Tween 80 (Sigma Aldrich, Saint Louis, MO, USA); Span 80 (Sigma Aldrich, Saint Louis, MO, USA); acetate and phosphate-buffered saline (ABS and PBS) were purchased from Sigma Aldrich, Saint Louis, MO, USA; MCF-10A cells were purchased from American Type Culture Collection, Manassas, VA, USA; DMEM/F12 supplemented with 5% horse serum (Sigma Aldrich, Saint Louis, MO, USA); 20 ng/mL EGF (Sigma Aldrich, Saint Louis, MO, USA); HDMVEC (Primary Dermal Microvascular Endothelial Cells) are from ATCC, Manassas, VA, USA; Milli-Q ultrapure distilled water (Merck, Rahway, NJ, USA). All other reagents used in this study were of analytical grade purity and were used without further purification. The human blood samples used were freshly obtained from one healthy nonsmoker volunteer.

### 2.2. Preparation Methods and Instrumentation

#### 2.2.1. CS-PEGA Synthesis

As reported in our previous study, we chemically modified CS with PEGA using Michael’s addition reaction under the same conditions [26,35]. To make CS-PEGA, CS (2.0 g) was dissolved in 100 mL of 1% (*w*/*w*) acetic acid solution in a 250 mL flask with a reflux condenser. Then, PEGA (molar ratio NH_2_:PEG = 1:0.5) was added slowly with a syringe through a rubber cap. The flask was filled with nitrogen (15 min), and the mixture was stirred for 48 h at 50 °C. After that, the flask was cooled to room temperature, and the pH of the mixture was raised to 8 with a NaHCO_3_-saturated solution. The polymer solution was then poured into acetone (500 mL), centrifuged, and washed with a lot of acetone (1.5 L). The product was collected and dialyzed against distilled water for 3 days. The final polymer was freeze-dried, and a light yellowish polymer with a reaction yield of 61% was obtained.

#### 2.2.2. Preparation of MPs/NPs Based on Chitosan Derivative

The method of Peptu et al. [17] was adapted to make chitosan micro/nanoparticles by a double crosslinking (ionic and covalent) process. The process used Na_2_SO_4_ and glutaraldehyde to cross-link the free NH_2_ of modified CS in a water-in-oil emulsion. The steps of making MPs/NPs were as follows: first, the water phase was made by dissolving CS-PEGA in 50 mL of 1% (*w*/*w*) CH_3_COOH (0.35%; 0.5%; 0.75% wt CS solutions). Then, the CS solution was mixed with a stabilizer Tween 80 (2% wt) for 5 min. Next, the oil phase was made by mixing toluene (200 mL) and another stabilizer Span 80 (2% wt) for 5 min. The water solution was then added slowly using a syringe (1 mL) to the organic phase under an Ultra Turrax (stirring speed 5000–15,000 rpm), forming the emulsion. Then, a fresh ionic crosslinker (Na_2_SO_4_, 5% wt) water solution was slowly added to crosslink the newly formed CS-PEGA particles for another 10 min. The formed emulsion was then moved to a reactor with a mechanical stirrer, where it was stirred for 1 h (500 rpm, room temperature) to complete the ionic crosslinking reaction. Next, the CS-PEGA particles were covalent crosslinked by adding to the emulsion glutaraldehyde (GA) dissolved in toluene (5 mL, c = 1.12 mg/mL) and stirred for another 1 h. Finally, the emulsion was centrifuged (1 h, 5000 rpm), the oil phase was removed, and the obtained MPs/NPs were resuspended in acetone. The excess reagents (Na_2_SO_4_, GA, and stabilizer agents) were then washed away from the MPs/NPs with water, acetone, and hexane.

#### 2.2.3. Determination of Structural and Morphological Characteristics of MPs/NPs

Fourier Transform Infrared Spectroscopy (FT-IR) spectra MPs/NPs and CS-PEGA were recorded with a DIGILAB SCIMITAR FTS 2000 spectrometer. The samples were prepared as KBr pellets and scanned over the wave number range of 4000–450 cm^−1^ at a resolution of 4.0 cm^−1^.

MPs/NPs morphology was investigated by SEM technique. SEM images were recorded with a HITACHI SU 1510 (Hitachi SU-1510, Hitachi Company, Tokyo, Japan) Scanning Electron Microscope. MP/NPs were fixed on an Aluminum stub and coated with a 7 nm thick gold layer using a Cressington 108 device before observation.

MPs/NPs’ mean diameter and size distribution were analyzed by laser diffractometry technique (SHIMADZU SALD 7001). MPs/NPs (3 mg) were immersed in acetone and sonicated for 15 min at room temperature using a sonication bath (Bandelin Sonorex). MPs/NPs suspension was added in a quartz cuvette equipped with a stirring mechanism and analyzed. All measurements were performed in triplicate.

MPs/NPs’ Zeta potential was analyzed with Zetasizer Nano ZS Series from Malvern. For sample preparation, MPs/NPs were dispersed in distilled water at a mass concentration of 1.25 g/L and sonicated for 1 min. Zeta potential measurements were performed at 25 °C.

#### 2.2.4. MP/NP Water Sorption Capacity

The swelling kinetics was investigated to determine the surface properties of the prepared MPs/NPs. Swelling kinetic results were observed gravimetrically and performed at pH 3.3 and 7.4 at room temperature. For the analysis of the water sorption capacity, pre-weighed MPs/NPs (0.03 g) were immersed in 1 mL ABS or PBS and maintained under mild stirring (100 rpm) at room temperature. After fixed time intervals, MPs/NPs were ultra-centrifugated (15,000 rpm), the supernatant was removed, and MPs/NPs were weighed. The process was repeated at different incubation time intervals continuously until the equilibrium swelling was achieved. The water uptake of the chitosan MPs/NPs was calculated by using the following equation:

(1) $$Q\%=\frac{w_{s}-w_{0}}{w_{0}}\times 100$$

where w_s_ is the weight of swollen MPs/NPs; w_0_ is the weight of dry MPs/NPs.

#### 2.2.5. MP/NP Drug-Loading Capacity

Levofloxacin (LEV) and ciprofloxacin (CIP), two antibiotics from the fluoroquinolone class, were used as model drugs for encapsulation into the MPs/NPs via a diffusional mechanism. In a typical procedure, MPs/NPs (0.03 g) were dispersed in 1 mL LEV or CIP aqueous solution (25 mg/mL) and sonicated for 15 min. MPs/NPs were incubated at room temperature, and mild stirring (100 rpm) for 3 days. After fixed time intervals, all MPs/NPs suspensions were filtered by ultra-centrifugation (15,000 rpm, 5 min), and 5 µL supernatant was withdrawn and replaced with fresh ABS. The supernatant was spectrophotometrically analyzed. The amount of entrapped LEV or CIP into MPs/NPs was calculated according to Equations (2) and (3), obtained based on a calibration curve, previously prepared.

(2) $$y=6.0424\cdot x;\;R^{2}=0.9973-LEV$$

(3) $$y=52877\cdot x;\;R^{2}=0.9972-CIP$$

Drug encapsulation efficiency (DEE) represents the added drug quantity (%) that is encapsulated into chitosan particles. The DEE was calculated according to Equation (4). The drug entrapment efficiency was determined with Equation (5).

(4) $$DEE=\frac{Actual\;drug\;\left(LEV\;or\;CIP\right)\;content\;loaded\;in\;MPs/NPs}{Theoretical\;drug\;content\;(LEV\;or\;CIP)}\times 100$$

(5) $$Entrapment\;efficiency=\frac{The\;total\;amount\;of\;LEV\;or\;CIP-The\;amount\;of\;LEV\;or\;CIP\;from\;supernatant}{The\;total\;amount\;of\;LEV\;or\;CIP}$$

Tests were performed with a spectrophotometer UV–VIS NanoDrop ND-1000. Drug-loaded MPs/NPs were lyophilized. The determinations are the average data of three replicates (N = 3).

#### 2.2.6. In Vitro Drug Release from MPs/NPs

The extent of particle swelling in aqueous media is primarily influenced by the internal architecture of the newly formed polymer network. The hydrophilic nature of MPs/NPs can provide valuable insights into predicting their drug loading/release behavior through the diffusion process. The in vitro drug release property of the LEV- or CIP-loaded MPs/NPs was assessed. The release process of LEV- or CIP-loaded MPs/NPs was conducted in a PBS medium (pH = 7.4), which ensured a slower release rate of both drugs. Lyophilized LEV- or CIP-loaded MPs/NPs (30 mg) were immersed in a 2 mL Eppendorf tube containing fresh PBS (1 mL) and maintained under continuous stirring (50 rpm). The suspension was then ultra-centrifuged (15,000 rpm, 5 min), and 5 µL of supernatant was withdrawn at fixed time intervals. The released LEV and CIP were analyzed spectrophotometrically at 287 nm (LEV) and 272 nm (CIP). The drug-releasing efficiency (Ref %) was calculated using Equation (6):

(6) $$Ref\left(\%\right)=\frac{m_{r}}{m_{l}}\times 100$$

where m_r_ is the amount of released drug (mg), m_l_ is the amount of drug loaded into chitosan MPs/NPs (mg).

#### 2.2.7. MPs/NPs Hemocompatibility

Hemolysis tests of MPs/NPs were conducted by adapting the Vuddanda et al. method [36]. Briefly, MPs/NPs were suspended in saline solutions at varying concentrations and combined with purified red blood cells (RBC, 2 mL, at a final concentration of 100, 250, 500 mg MPs/NPs/mL). Positive (100% lysis) and negative (0% lysis) control samples were prepared by adding equal volumes (2 mL) of Triton X-100 and normal saline solution (PBS). The samples were incubated at 37 °C for 2, 4, and 6 h and gently shaken every 30 min for re-homogenization of erythrocytes and MPs/NPs. After a pre-established time, the samples were centrifuged (2000 rpm, 5 min), and 1.5 mL of supernatant was incubated for another 30 min at room temperature to allow hemoglobin oxidation. The oxyhemoglobin was spectrophotometrically analyzed at 540 nm. Hemolysis was calculated using Equation (7). All samples were analyzed in triplicate, and the results were averaged.

(7) $$\%Hemolysis=\frac{{Absorbance}_{sample}-{Absorbance}_{negativecontrol}}{{Absorbance}_{pozitivecontrol}-{Absorbance}_{negativecontrol}}\times 100$$

where

The absorbance of the negative control is the absorbance of PBS;

The absorbance of the positive control is the absorbance of Triton X-100.

#### 2.2.8. MPs/NPs Cytotoxicity

##### Cell Culture

All cells were cultured in a humidified atmosphere at 37 °C with 5% CO_2_. Moreover, 4T1 mouse breast carcinoma cells (ATCC, CRL-2539) and MDA-MB-231 human breast epithelial carcinoma cells (ATCC) were cultured as described by Gjerdrum et al. [37] MCF-10A (ATCC) (a gift from Prof. Dr. J. Lorens) were maintained in DMEM/F12 supplemented with 5% horse serum (Sigma Aldrich, Saint Louis, MO, USA), 20 ng/mL EGF (Sigma Aldrich, Saint Louis, MO, USA), 10 ug/mL insulin (Sigma Aldrich, Saint Louis, MO, USA), 0.5 ng/mL hydrocortisone (Sigma Aldrich, Saint Louis, MO, USA), 10 ng/mL cholera toxin (Sigma Aldrich, Saint Louis, MO, USA), 1% Pen/Strep (Sigma Aldrich, Saint Louis, MO, USA); A549 (ATCC) were cultured in F-12K Medium, supplemented with100 U/mL of penicillin and 100 μg/mL of streptomycin and 5% bovine serum (Sigma Aldrich Aldrich, Saint Louis, MO, USA). Cell viability was measured in 4 wells for each treatment at 72 h from starting the treatment. CellTiter-Blue^®^ Cell Viability Assay (Promega, Madison, WI, USA) has been used to investigate cell viability. Measurements have been performed using a multi-plate reader (FilterMax F5).

## 3. Results and Discussion

Our study employed a CS-modified PEGA to enhance the water solubility of CS. The structural characteristics of the prepared CS-modified PEGA were essentially similar to those previously reported. Further studies aim to establish the amenability of CS-PEGA for an ionic crosslinking process in the presence of Na_2_SO_4_, followed by covalent crosslinking. Although TPP has been widely employed in such processes, more research is needed to compare the properties and applications of CS-PEGA crosslinked with Na_2_SO_4_.

### 3.1. Preparation of MPs/NPs Based on Chitosan Derivative

Our purpose in preparing prepared MP/NP-based CS-PEGA was to obtain a new drug delivery system with final properties that can be modulated through the modification of initial process parameters such as the water/oil phase ratio, stirring speed, and nature of ionic crosslinker. Moreover, in the present study, we were interested in investigating the change of the ionic crosslinker from TPP to sodium sulfate and its influence on the final products. Further, another goal was for the prepared system to present the capacity to entrap and release different types of drugs, thus having the ability to be used for various biomedical applications. The created system demonstrated great potential in replacing the administration of antibiotics in their “free” form.

The proposed method for synthesizing MPs/NPs involves a dual crosslinking technique in water-in-oil emulsion (w/o). The process is based on the idea of creating a network that is linked by both ionic and covalent bonds. The first stage involves mostly ionic crosslinking, while the second stage involves covalent crosslinking. The major problem correlated with the crosslinking mechanism is the effect of CS chemical modification on amine group availability for further reactions. The ionic crosslinking process takes place due to the interaction between CS positively charged amino groups and negatively charged sulfate groups of sodium sulfate. Likewise, the covalent crosslinking reaction with GA takes place at the amino groups from the CS chain, with the formation of imine bonds. The advantage of the selected method is the use of a lower quantity of covalent cross-linkers, which leads to a final product with low toxicity. The prepared MPs/NPs were evaluated based on several parameters, including size and polydispersity, morphology, drug loading and release, Zeta potential, hemocompatibility, and cytotoxicity analysis. Table 1 highlights the experimental program concerning the preparation parameters, size, and Zeta potential of MPs/NPs. It is shown that the key parameters were polymer concentration, NH_2_/Na_2_SO_4_ molar ratio, and stirring speed in the emulsion formation phase. The other initial parameters (water/oil phase ratio, stabilizer concentration, and GA content) were fixed throughout the study.

**Figure 1:**
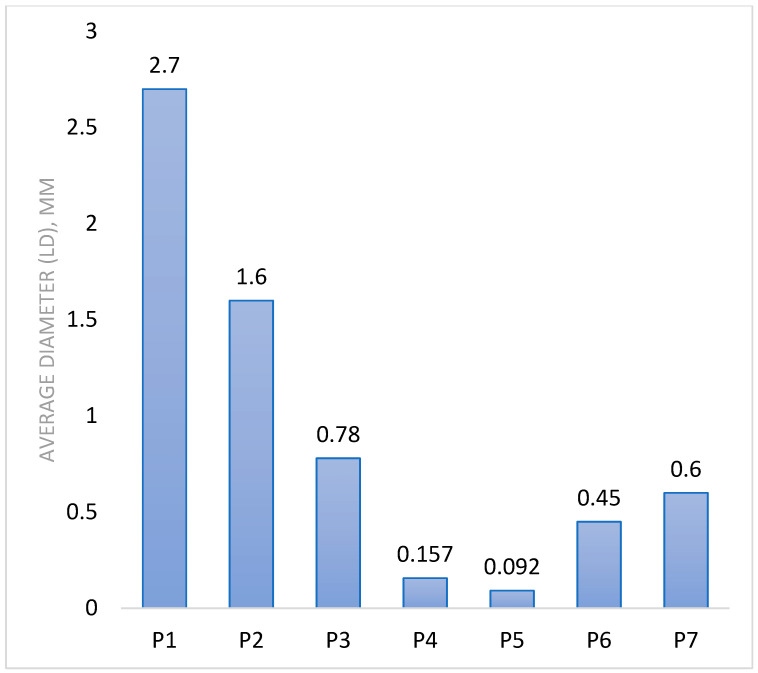
MPs/NPs average diameter variation, µm.

To investigate the effect of different parameters on the final properties of MPs/NPs, we varied the polymer concentration, the molar ratio (NH_2_/Na_2_SO_4_), and the stirring speed. The obtained results indicate that all three studied parameters influenced the final size diameter of the prepared particles, respectively, the swelling and loading/releasing drug capacity. Moreover, the employed amounts of ionic crosslinker were established through separate experiments, and lower values do not provide enough crosslinking (sample P0), while higher values (sample P8) do not influence the final properties of the micro and nanoparticles morphology, suspension stability, swelling degree, and drug loading/releasing capacity. Thus, we took into consideration for discussion only the P1-P8 samples. The SEM images and granulometry measurements confirmed that spherical MPs/NPs were obtained, and the use of a lower polymer concentration resulted in smaller particle sizes. For instance, switching the initial polymer concentration from 0.5% (P4) to 0.35% (wt) (P5) led to a decrease in particle size from 157 nm to 92 nm (Table 1 and Figure 1). A lower polymer concentration leads to a decrease in the viscosity of the solution, thereby resulting in the formation of smaller droplets and nanometer-sized particles.

**Figure 2:**
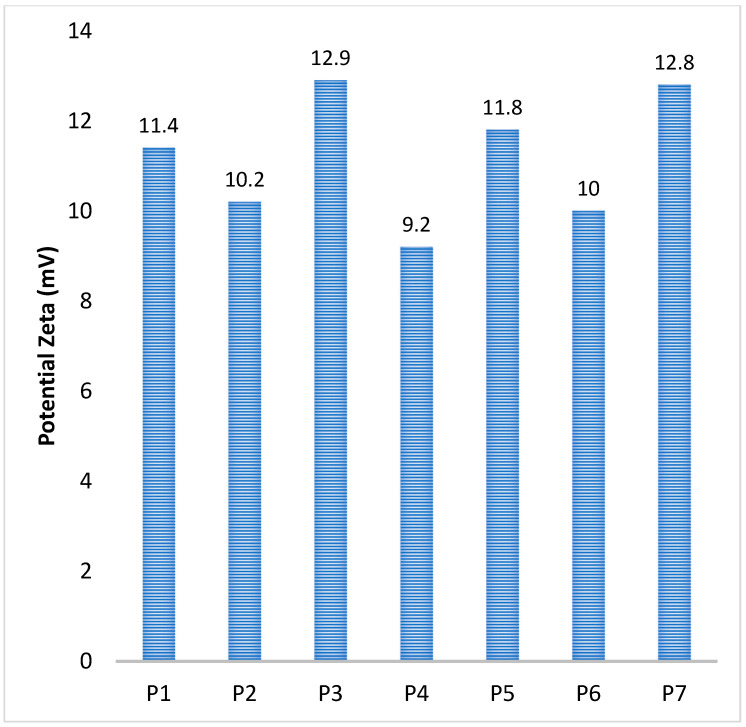
MPs/NPs Zeta potential values.

When the emulsification speed was ramped up from 5000 rpm (P1) to 15,000 rpm (P4), it led to a reduction in particle size from 2700 nm down to 157 nm. This is because a faster emulsification speed contributes to a superior dispersion, which in turn facilitates the creation of smaller droplets within the emulsion phase. Also, an increase in the quantity of the ionic crosslinker (Na_2_SO_4_) is associated with a rise in particle size from 157 nm (P4) to 450 nm (P6). This outcome aligns with expectations, as an elevated amount of ionic cross-linker introduces more available sulfate groups. This leads to a faster ionic gelation, which subsequently results in an enlargement of the MPs/NPs particle size [38]. Zeta potential measurements were conducted to analyze the surface charge of MPs/NPs samples and, implicitly, their stability against agregation. The condition of a particle system to be stable and to not form clumps or aggregates in water is a significant negative or positive value of Zeta potential. The information in Table 1 and Figure 2 shows that MPs/NPs have slightly positive values, which are lower than those typically associated with pristine CS. Despite this, they maintain good stability in water due to electrostatic repulsions. The Zeta potential values of the particles can be linked to the limited availability of NH_2_ groups. These groups are grafted with PEGA and also take part in the covalent crosslinking process. This suggests that these slightly positive values, while lower than pristine CS, are still effective in ensuring stability in an aqueous suspension.

### 3.2. Structural and Morphological Characteristics of MPs/NPs

**Figure 3:**
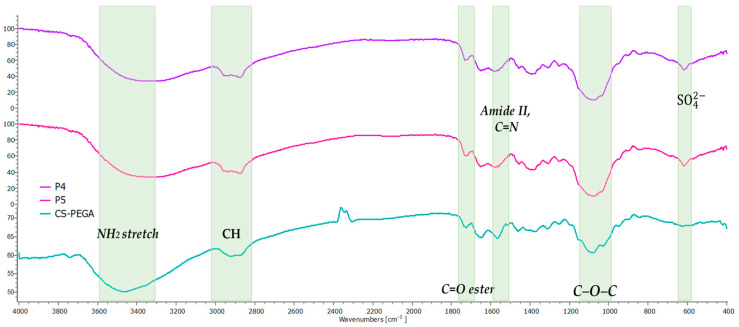
FTIR spectra of sample P4 and CS-PEGA.

FTIR spectroscopy has been used to determine the functional groups of MPs/NPs. The FTIR analysis revealed a similar profile for all MPs/NPs and confirmed the successful crosslinking process by the presence of characteristic groups of both crosslinking processes, thus only the FT-IR spectra for samples P4 and P5 which presented the optimum morphology properties are displayed (Figure 3). Table 2 presents in detail the characteristics absorption band as an example for sample P4. The first crosslinking process confirmed by FTIR was the ionic one, the spectra revealing at 617 cm^−1^ the presence of a new bond formed between SO_4_^2−^ anions of Na_2_SO_4_ and NH_3_^+^ cations from CS-PEGA. The absorption band observed at 1575 cm^−1^ is assigned to the covalent crosslinking process between CS and GA [26,35,39,40,41,42].

**Figure 4:**
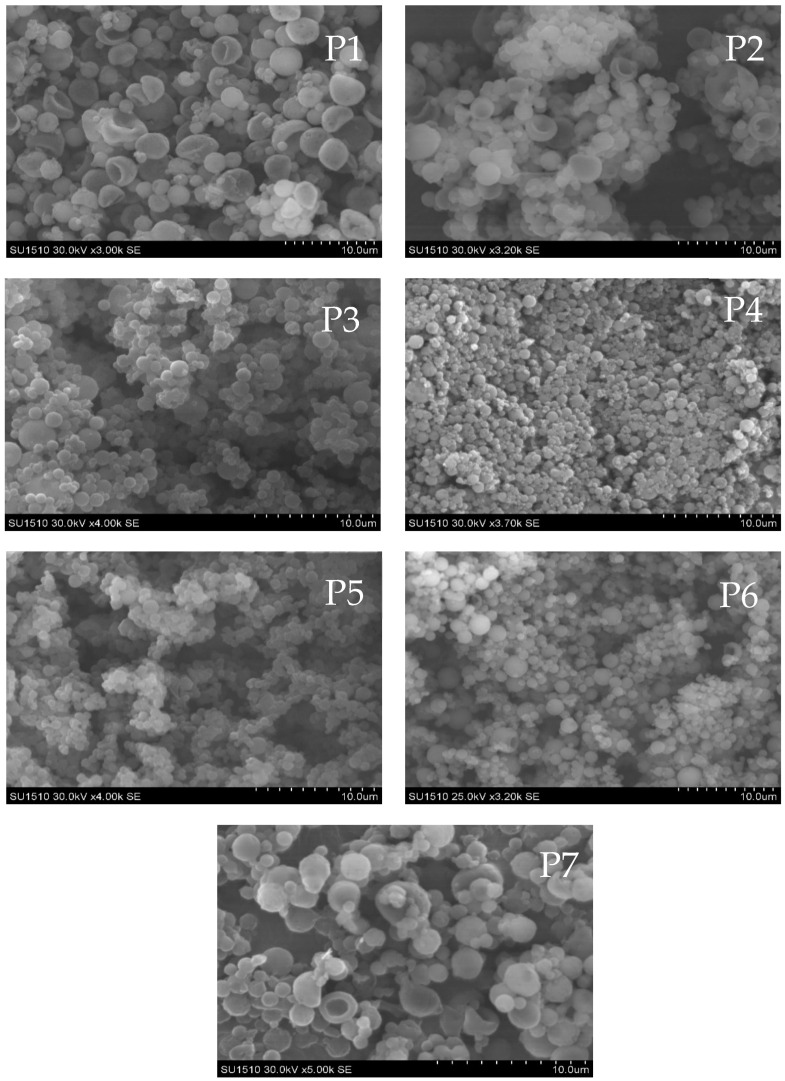
Micrograph SEM of optimized MPs/NPs ionically crosslinked with Na_2_SO_4_ (magnification graphical bar length P1–P7: 0.01 mm).

Further SEM techniques were employed to determine the external morphology of MPs/NPs and reveal whether the particles are spherical, smooth, or rough in their surfaces. Considering the main purpose of the study, which is to use the particle as a potential drug delivery system, they must exhibit certain characteristics such as nanometric dimensions, stability, and individuality. Figure 4 depicts SEM images for all synthesized samples. Analyzing the recorded pictures revealed that MPs/NPs are characterized by a spherical shape, are well individualized, and have reduced diameter and polydispersity. Moreover, it may be observed that all MPs/NPs samples are homogeneous, separated, and without impurities. Given the optimization steps of the MPs/NPs synthesis, SEM-recorded images revealed important morphological differences in terms of particle size, which is dependent on the polymer concentration and stirring speed. Therefore, lowering the aqueous phase concentration led to particles with lower dimensions. Similarly, results were obtained when the stirring speed was raised from 5000 rpm to 15,000 rpm. A visual comparison between samples P1 and P4 (Figure 4) confirms the size reduction. SEM images revealed that the particle size of MPs/NPs is influenced by the concentration and nature of the crosslinking agent. Specifically, using a higher concentration of Na_2_SO_4_ increases the intra-molecular reactions, leading to an increase in particle size. This observation is consistent with the results obtained when hydrogel particles based on CS-PEGA using sodium tripolyphosphate (TPP) as an ionic crosslinking agent were synthesized in our previous study. Furthermore, a difference in particle size was observed when the ionic crosslinker was modified. Our previous results showed that when TPP was used as an ionic cross-linker, the particle size was smaller compared to the particle size when Na_2_SO_4_ was used. The observed tendency could be explained since particles crosslinked with Na_2_SO_4_ may present a reduced crosslinking density, which leads to a more loose polymer network, compared with particles crosslinked with TPP, which present a denser polymer network and, subsequently, smaller particles.

**Figure 5:**
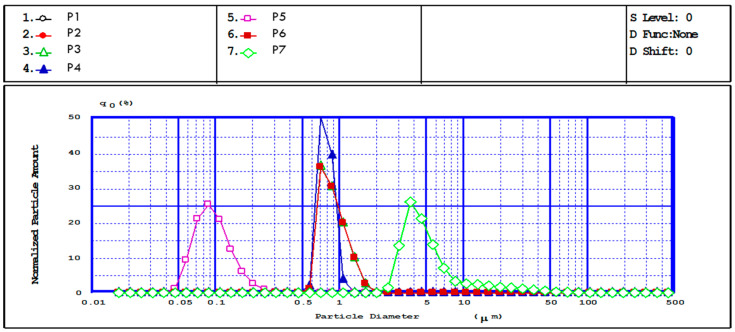
Granulometric distribution curves of P1–P7 samples.

Laser diffraction is an important method for characterizing particle size in solution (Figure 5). The analysis was performed in acetone, which is a poor solvent for CS, thus avoiding the sample’s swelling. The data results were in good agreement with SEM observations and confirmed the particle size dependence on initial synthesis parameters such as polymer solution concentration and stirring speed. Higher emulsification stirring speed leads to improved dispersion and also to the generation of small droplets in the emulsion system, resulting in nanoparticles. MPs/NPs granulometric distribution curves (determined by laser beam diffraction (LD), Figure 5 and Table 1) have an unimodal aspect, and analyzed samples present a nanometric diameter, depending on the initial preparation conditions. In the case of samples P1–P4, the stirring speed was increased from 5000 to 15,000 rpm/min, and a size reduction was observed due to better dispersion of the emulsion phase. These results are consistent with earlier published data for CS hydrogel particles [26].

**Figure 6:**
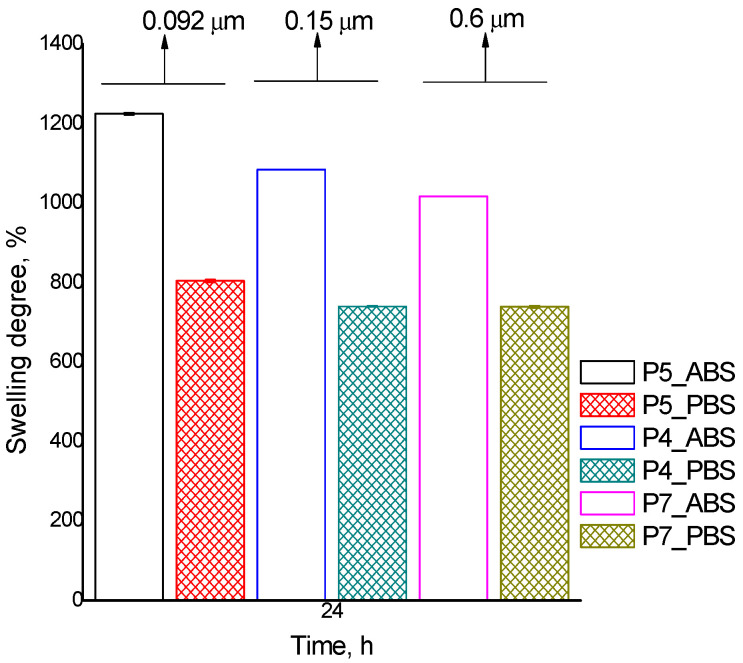
Polymer concentration influence on the swelling process for P5 (0.35%); P4 (0.5%); P7 (0.75%).

**Figure 7:**
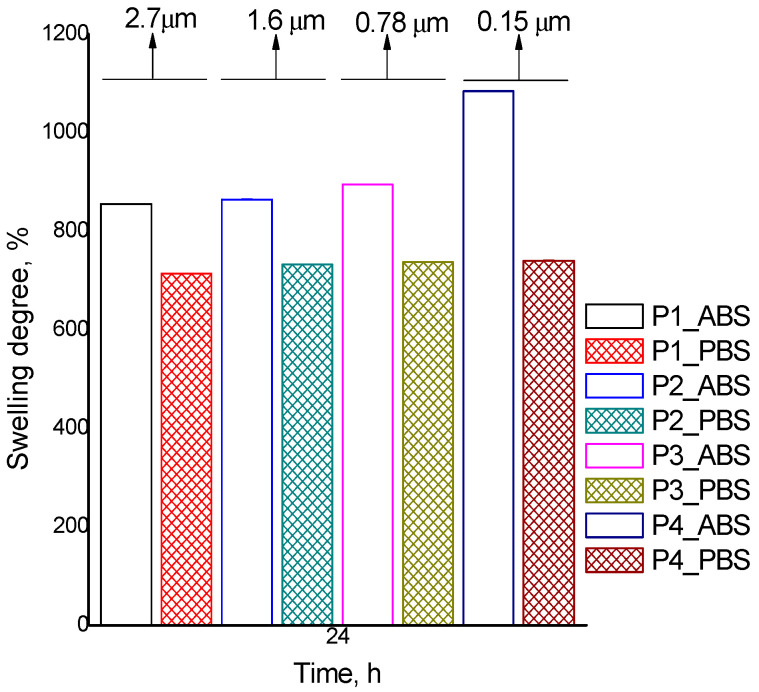
Stirring speed influence on the swelling process for MP/NP samples: P1 (5000 rpm); P2 (9000 rpm); P3 (12,000 rpm); P4 (15,000 rpm).

The swelling ability of nanoparticles is generally associated with free hydrophilic groups and surface properties. Particle swelling behavior in aqueous media is dependent on the nature of both the solvent and polymer (mainly on the nature of the internal architecture of the newly formed polymer network). The ability of nanoparticles to take up water in different physiological fluids is fundamental for their use in biomedical applications. Thus, to explore MPs/NPs interaction with aqueous environments, swelling studies were performed gravimetrically in acidic (pH = 3.3) and neutral (pH = 7.4) media. The results from Table 3, Figure 6 and Figure 7 allow us to ascertain that prepared MPs/NPs present good swelling ability in both aqueous environments. However, as expected when using acidic conditions (ABS), a higher swelling degree (1225%, sample P5) was observed compared to the neutral environment (PBS) (804%, sample P5). The mentioned effect is determined by the -NH_2_ groups of the CS-PEGA, which did not participate in the dual crosslinking process. In acidic conditions, CS amine groups are protonated, leading to electrostatic repulsion between the ammonium cations formed. The immediate effect is an increase in the distance between the network chain and its meshes, leading to higher water uptake into the polymer network. However, the swelling degree values for neutral conditions are also quite high but lower compared to acidic conditions because the protonation of -NH_2_ groups of the CS derivative is no longer possible. Therefore, H-bonds are formed into the polymer network. The study examined the influence of polymer concentration and stirring speed on the swelling properties of MPs/NPs (Figure 6 and Figure 7). The results revealed that the swelling degree values of MPs/NPs are dependent on the polymer concentration and stirring speed. The data showed that a lower polymer solution concentration and a higher stirring speed lead to a higher MPs/NPs capacity to retain water. The highest swelling degree value for both acidic and neutral conditions was obtained for sample P5, which had a low polymer solution concentration (0.35%) and a higher stirring speed (15,000 rpm). A possible explanation for this behavior could be due to an increased agglomeration tendency, which is also confirmed by SEM images and a lower crosslinking density of the polymer matrix. The results also showed that a lower stirring speed and a higher concentration of polymer solution led to a decrease in the amount of retained water (samples P1 and P7). Moreover, the obtained results show that the sample’s water absorption depends on the polymer molecule’s ability to link together to form the network. The more crosslinked the samples are, the less water they absorb [43,44].

Also, when the amount of Na_2_SO_4_ was enhanced (in fact of the molar ratio sodium sulfate/CS), it was observed that the swelling degree value slightly decreased. This is a logical consequence due to a better network crosslinking density. Based on these results, it can be concluded that MPs/NPs ionically crosslinked with Na_2_SO_4_ presented a higher water absorption capacity compared with particles crosslinked with TPP, data published in our previous study. A possible explanation for this behavior may be considered to be an increase in surface hydrophilicity of the particles or a lower crosslinking degree, which leads to possible larger meshes of the polymer network and, hence, a higher water absorption ability. Considering the obtained results, it can be concluded that the presence of CS-PEGA in the composition of MPs/NPs plays an important role and confers them to a pH-sensitive character.

MP/NP stability in aqueous suspension was analyzed by Zeta potential measurements. The Zeta potential represents a physical property displayed by all particle categories in suspension. This property highlights the NP stability in suspensions depending on the NP surface charge. According to the literature studies, particles that exhibit a Zeta potential of above +30 mV or below −30 mV express adequate electrostatic repulsion to provide colloidal stability [45]. Table 1 summarizes the arithmetic average of Zeta potentials determined for MPs/NPs. The analyzed dried samples were dispersed in distilled water (c = 1.25 g/L) and sonicated for 15 min to avoid agglomeration. The determinations were performed in triplicate at 25 °C. The obtained results showed that all prepared samples presented good stability in aqueous suspension and were positively charged with a Zeta potential varying between 9.2 ± 0.3 and 12.9± 0.6.

The ability of MPs/NPs to entrap active principles were analyzed using two broad-spectrum antibiotics, levofloxacin and ciprofloxacin, which belong to the class of fluoroquinolones and are generally used to treat antibacterial infections. Levofloxacin and ciprofloxacin are hydrophilic drugs and are suitable candidates for designed particulate drug delivery systems used for distinct biomedical applications to reduce the frequency of drug administration [46]. The incorporation process of LEV or CIP into all samples was performed by a diffusion process. Thus, the LEV and CIP content in the supernatant was determined spectrophotometrically (at 287 nm for LEV and 272 nm for CIP). The results showed that the MPs/NPs were able to encapsulate between 1.61 and 2.05 mg LEV/mg particles and 1.62 and 1.67 mg CIP/mg particles (Table 4). Therefore, the maximum drug loading efficiency value obtained for LEV was 87.0% and 99.99% for CIP, thus showing a high capacity of the micro/nanoparticulate polymer support to retain biologically active compounds. Table 4 highlights the obtained results regarding the ability of MPs/NPs to encapsulate LEV or CIP. The encapsulation efficiency of LEV and CIP was calculated using Equations (4) and (5). MPs/NPs DEE presented high values. The experimental results are in agreement with the MPs/NPs swelling ability previously discussed. It is important to highlight that due to the enhanced capacity of MPs/NPs to absorb water, the obtained values for drug encapsulation are higher compared to the experimental data previously reported.

**Figure 8:**
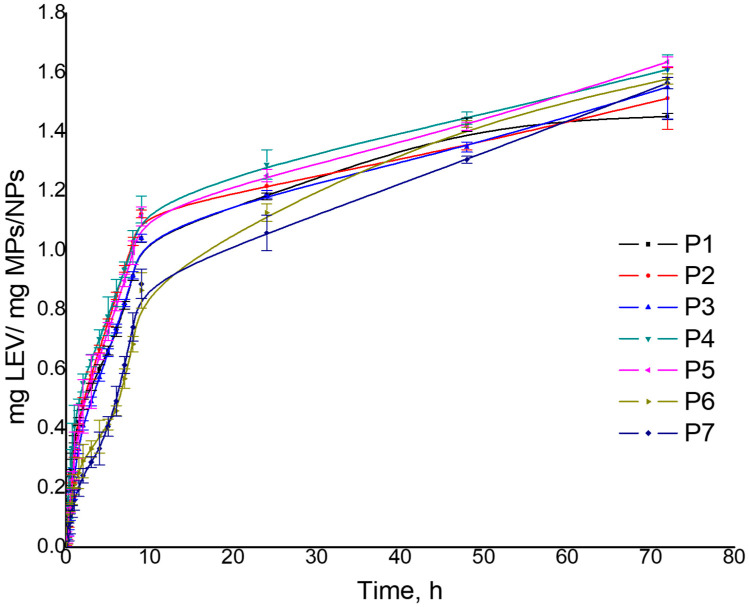
MPs/NPs’ in vitro LEV release.

**Figure 9:**
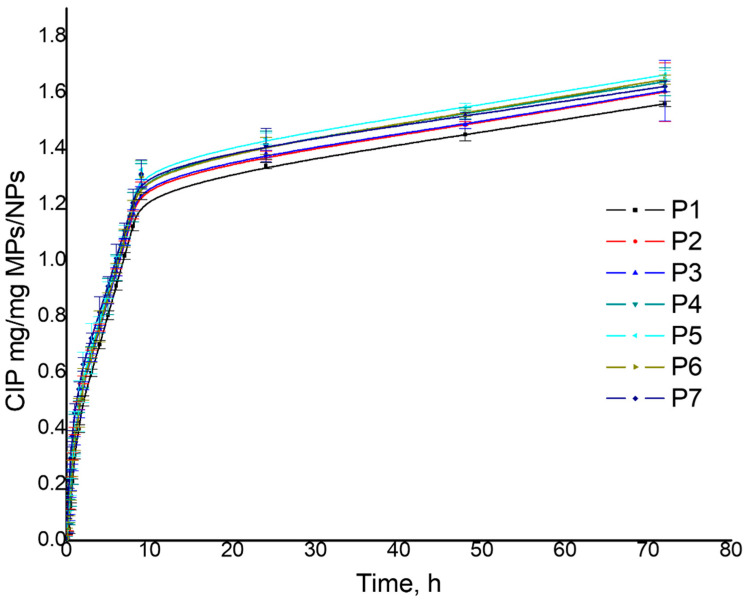
MPs/NPs’ in vitro CIP release.

The release capacity of LEV or CIP from MPs/NPs was analyzed by the diffusion technique in an aqueous medium (pH = 7.4) at 37 °C. Table 5 presents the obtained results for MP/NP drug release efficiency. The obtained results showed that the LEV and CIP release process present a fast phase that is reached within 9 h, followed by a slower release phase (characterized by a constant release) until 72 h. The sustained release of LEV and CIP from MPs/NPs is due to both drugs being adsorbed in the depth of the MPs/NPs network, which has excellent swelling properties. Also, considering the influence of initial preparation parameters on the release ability of the MPs/NPs, similar behavior as for drug loading was observed. The maximum released amount of LEV varied between 1.45 and 1.63 mg/mg MPs/NPs, respectively, and 1.55 and 1.63 mg CIP/mg MPs/NPs (Table 5, Figure 8 and Figure 9). Moreover, the LEV release efficiency for all samples presented values between 79 and 96%, respectively, and 96 and 99% for CIP release efficiency. The highest value of drug release efficiency was noticed for sample P5, which has also shown a high capacity of water absorption.

#### Theoretical Modeling of Drug Release

**Figure 10:**
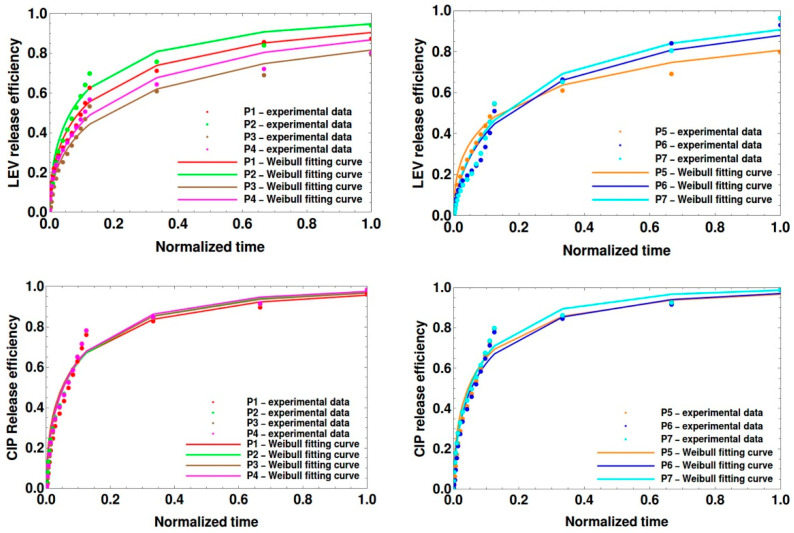
The experimental data and the Weibull theoretical curve for levofloxacin (first row) and ciprofloxacin (second row).

There are many equations used to model the drug release from polymeric matrices, each applied best on different phases of the release: for the burst phase, the Higuchi equation; for the swelling phase, the Korsmeyer–Peppas equation; for the equilibrium phase, the Peppas Sahlin equation [47,48]. All these are small-order approximations of series expansion for the Weibull exponential equation:

(8) $$\frac{M_{t}}{M_{\infty }}=1-e^{{-at}^{b}}$$

where M_t_, $M_{\infty }$ represent the amount released at the time t, respectively, as the time approaches infinity, a and b constants, of which b values indicate the drug release mechanism: the Fickian diffusion if b is smaller than 0.75, combined release, i.e., diffusion and chain relaxation; for b values between 0.75 and 1, complex release, i.e., diffusion and chain relaxation and degradation; for b values higher than 1 [49]. The experimental release kinetics (Figure 7) showed that the drug release is in the swelling phase, close to the equilibrium one. The fitting of experimental results, normalized, on the Weibull equation offered the best results, in terms of correlation factor, compared to Korsmeyer–Peppas and Peppas Sahlin equations (Figure 10).

The Weibull parameters, a and b, are given in Table 6; for easier observation of possible dependencies, the synthesis parameters, as well as the particle size, were also included.

One first observation is that b values are smaller than 0.75, indicating that, in the observation interval, the releases are dominated by Fickian diffusion for all samples and both levofloxacin and ciprofloxacin. Moreover, they are close as values for both drug types, suggesting that they are not decisively influenced neither by the polymer network or drug type but, more likely, by the release time: if release is prolonged to long time intervals, phenomena like polymer relaxation and degradation appear and b value will increase over the threshold values of 0.75 and 1. Even if the Weibull equation offers very good results in modeling the drug release [49,50,51,52], it has the disadvantage of being a statistical equation, not based on the physico-chemical phenomena taking place in the drug release system, limiting thus their understanding. The use of Scale Relativity Theory (SRT) reduces this shortcoming by offering constants a and b physical significance. TRS implies that the entire drug release system, i.e., polymeric particle, drug, water, is considered a medium without interactions by equating the system complexity with fractality, for which the drug molecules are released on fractal curves, characterized by a fractal dimension D_F_ as a measure of system nonlinearity and complexity [53,54]. In this theory, the Weibull equation is obtained as a short-time approximation of motion when convective processes are dominant [55], and the Weibull parameters can be defined as

(9) $${\left(\frac{n\pi }{L}\right)}^{2}=\frac{D}{\Gamma \left(\frac{2}{D_{F}}+1\right)}$$

$$b=\frac{2}{D_{F}}$$

where n is the diffusion order, D is a structure coefficient that can be identified with the diffusion coefficient, L is a system characteristic length, and $\Gamma =\left(\frac{2}{D_{F}}\right)=\int_{0}^{\infty }x^{\left(\frac{2}{D_{F}}\right)-1}e^{-x}dx$.

We highlight that thus dependences of Weibull parameters on system complexity and nonlinearity (through fractal dimension D_F_), on system size (through system characteristic length L), and diffusion coefficient are introduced. Considering the diffusion order n = 1 (since it is a Fickian diffusion) and assimilating the system characteristic length with the particle diameter d, the diffusion coefficient and the fractal dimension of drug molecule trajectories can be calculated using:

(10) $$D={\left(\frac{L}{n\pi }\right)}^{2}\cdot a\cdot \Gamma \left(b+1\right)$$

$$D_{F}=\frac{2}{b}$$

and the values are presented in Table 7.

An analysis of the obtained values led us to the conclusion that system complexity/nonlinearity and release mechanism are dominantly polymer-dependent rather than drug-dependent (fractal dimension and b Weibull parameter have close values for each polymer, regardless of the drug considered), while the diffusion coefficient is influenced by both the nanoparticle size/structure and the drug.

### 3.3. Biological Properties of MPs/NPs

**Figure 11:**
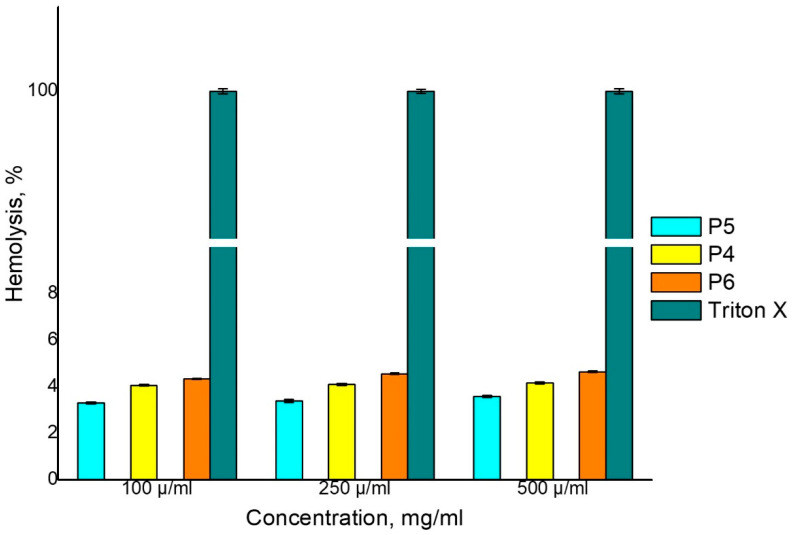
Influence of NPs on the Hemolysis Degree.

The hemolytic potential of MP/NP-based CS-PEGA has been studied. The hemolysis test is a mandatory requirement for blood-borne materials since their interaction with blood components may lead to erythrocyte lysis. For this reason, the effects of the prepared NPs on the blood were evaluated using a hemolysis assay. The results obtained from this study revealed that the degree of hemolysis increases with the increase of the NPs concentration (Figure 11). It has been found that the NPs prepared present very good compatibility with the normal blood (<10% compared to the positive control) [56]. Also, a 1% increase in hemolysis percentage for the P6 sample was observed in this case using an increased quantity of ionic crosslinker Na_2_SO_4_.

**Figure 12:**
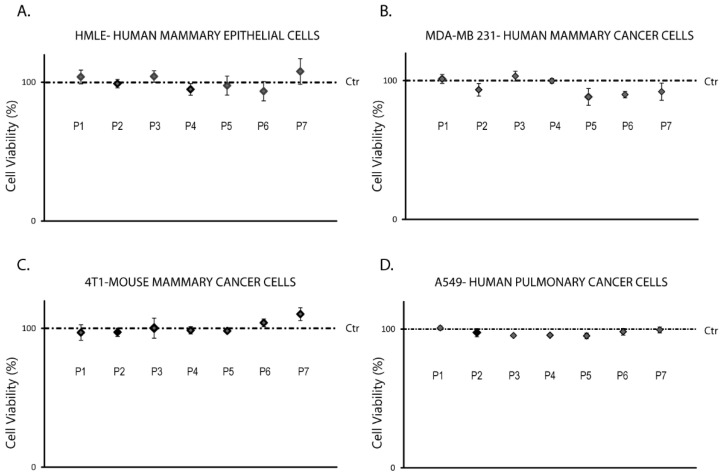
Cell viability. (**A**). HMLE-Human mammary epithelial cells. (**B**). MDA-MB 231-Human mammary cancer cells. (**C**). 4T1-Mouse mammary cancer cells. (**D**). A549-Human Pulmonary cancer cells.

The viability of HMLE-Human mammary epithelial cells (normal mammary cell line), MDA-MB 231-Human mammary cancer cells, 4T1-Mouse mammary cancer cells, and A549-Human Pulmonary cancer cells, respectively, in 2D culture treated with micro and nanoparticulated formulations, was not affected by the presence of ionic crosslinker in the MPs/NPs structure (Figure 12).

## 4. Conclusions

This research successfully demonstrates the development of a micro/nanoparticulated system. This system is based on a chitosan derivative that has been grafted with PEGA. CS-PEGA micro/nanoparticles were prepared using a dual crosslinking technique (ionic followed by covalent) applied in a reverse emulsion system. The MPs/NPs’ final properties can be modulated through the modification of initial process parameters such as the water/phase ratio, stirring speed, and nature of the ionic crosslinker. SEM analysis revealed that MPs/NPs are spherical, homogeneous, and have submicronic dimensions. Moreover, the average size of MPs/NPs, analyzed via laser diffractometry, ranges from 2700 to 92 nm, depending on specific preparation conditions, in agreement with SEM images. The swelling properties of MPs/NPs were examined in two distinct environments: acidic (pH = 3.3) and basic (pH = 7.4). The results showed a higher water absorption capacity in the acidic medium compared to the basic one. This suggests that the MPs/NPs are more responsive to an acidic environment. Furthermore, it was found that the swelling abilities of MPs/NPs are directly affected by the initial process parameters, which are correlated with particle size. This implies that careful control of the process parameters can influence the swelling behavior and, consequently, the performance of the MPs/NPs. The MP/NP-based CS-PEGA potential as drug-delivery devices for biomedical applications was demonstrated via in vitro release experiments using levofloxacin and ciprofloxacin as model drugs. MPs/NPs showed good diffusional loading and releasing capacity. Hemocompatibility analysis results showed a hemolysis percentage of less than 10%, indicating their hemocompatibility. Cytocompatibility studies demonstrated that MPs/NPs based CS-PEGA do not significantly affect normal and cancer cell toxicity. Overall, the studies presented demonstrate the feasibility of preparing a micro/nanoparticulate system that is nontoxic and biocompatible, with potential applications as drug delivery devices. This innovative approach could potentially open up new avenues in the field of nanoparticle research and application.

## Figures and Tables

**Table 1:** MPs/NPs–Experimental program preparation, size, and Zeta potential measurements.

Sample Code	Polymer Solution Concentration, %	Molar Ratio NH_2_/Na_2_SO_4_	Speed, rpm	Water Phase, mL	The Organic Phase, mL	Surfactants Concentration, %	Ionic/Covalent Crosslinking Time, h	Average Diameter (LD), µm	Potential Zeta (mV)
P0	0.5	1:3	5000	50	200	2	1	-	-
P1	0.5	1:4	5000	50	200	2	1	2.7	11.4 ± 0.2
P2	0.5	1:4	9000	50	200	2	1	1.6	10.2 ± 0.3
P3	0.5	1:4	12,000	50	200	2	1	0.78	12.9 ± 0.6
P4	0.5	1:4	15,000	50	200	2	1	0.157	9.2 ± 0.3
P5	0.35	1:4	15,000	50	200	2	1	0.092	11.8 ± 0.6
P6	0.5	1:5	15,000	50	200	2	1	0.45	10 ± 0.4
P7	0.75	1:4	15,000	50	200	2	1	0.6	12.8 ± 0.3
P8	0.5	1:6	15,000	50	200	2	1	0.45	15 ± 0.4

**Table 2:** Characteristic absorption bands of sample P4 and P5.

Sample	Wavelength (cm^−1^)	Absorption Band
P4 and P5	617	The new bond formed between the SO_4_^2−^ anions and NH_3_^+^ cations (ionic crosslinking process)
	1086	Stretching vibrations -C-O-C
	1575	Imine linkages -C=N-

**Table 3:** Swelling degree of P1–P7.

Sample Code	P1	P2	P3	P4	P5	P6	P7
Q _ABS_, %	855	864	894	1084	1225	1046	1017
Q _PBS_, %	714	732	737	740	804	749	739

**Table 4:** Chitosan MP/NP capacity to encapsulate LEV and CIP.

Sample Code	P1		P2		P3		P4		P5		P6		P7	
Drug	LEV	CIP	LEV	CIP	LEV	CIP	LEV	CIP	LEV	CIP	LEV	CIP	LEV	CIP
mg LEV/CIP/mg MPs/NPs	1.663	1.617	1.609	1.627	1.956	1.633	2.010	1.665	2.054	1.666	1.698	1.666	1.624	1.638
Encapsulation efficiency, %	82.18	99.96	84.68	99.92	84.52	99.92	87.5	99.90	87.9	99.98	85.94	99.96	83.15	99.94

**Table 5:** Released drug and efficiency.

Sample Code	P1		P2		P3		P4		P5		P6		P7	
Drug	LEV	CIP	LEV	CIP	LEV	CIP	LEV	CIP	LEV	CIP	LEV	CIP	LEV	CIP
mg LEV/CIP/mg MPs/NPs	1.452	1.560	1.513	1.602	1.552	1.605	1.610	1.639	1.637	1.663	1.579	1.646	1.565	1.621
Release efficiency, %	87.29	96.43	93.98	98.43	79.30	98.33	80.25	98.42	79.68	99.81	92.95	98.80	96.35	98.94

**Table 6:** Weibull parameters.

Sample Code	Polymer Solution Concentration (%)	Molar Ratio NH_2_/Na_2_SO_4_	Speed (rpm)	Average Diameter (µm)	LEV		CIP	
					a	b	a	b
P1	0.5	1:4	5000	2.7	2.36	0.51	3.16	0.5
P2	0.5	1:4	9000	1.6	2.97	0.53	3.58	0.56
P3	0.5	1:4	12,000	0.78	1.7	0.51	3.44	0.53
P4	0.5	1:4	15,000	0.157	2.03	0.53	3.73	0.57
P5	0.35	1:4	15,000	0.092	1.65	0.44	3.44	0.51
P6	0.5	1:5	15,000	0.45	2.12	0.61	3.58	0.56
P7	0.75	1:4	15,000	0.6	2.39	0.64	4.43	0.61

**Table 7:** The values for fractal dimensions and diffusion coefficients of drug.

Sample Code	Average Diameter (µm)	LEV		CIP	
		$D\left(\frac{µm^{2}}{s}\right)$	D_F_	$D\left(\frac{µm^{2}}{s}\right)$	D_F_
P1	2.7	1.547	3.92	2.07	4.00
P2	1.6	0.684	3.77	0.826	3.57
P3	0.78	0.093	3.92	0.188	3.77
P4	0.157	0.004	3.77	0.008	3.51
P5	0.092	0.001	4.54	0.002	3.92
P6	0.45	0.039	3.28	0.065	3.57
P7	0.6	0.078	3.13	0.144	3.28

## Data Availability

Data are contained within the article.
